# Integrated Analysis of Key Genes and Pathways Involved in Fetal Growth Restriction and Their Associations With the Dysregulation of the Maternal Immune System

**DOI:** 10.3389/fgene.2020.581789

**Published:** 2021-01-08

**Authors:** Xue Wang, Hong Zhu, Lei Lei, Yang Zhang, Chao Tang, Jia-xing Wu, Jie-ru Zhou, Xi-rong Xiao

**Affiliations:** ^1^Despartment of Obstetrics and Gynecology, Xin Hua Hospital Affiliated to Shanghai Jiao Tong University School of Medicine, Shanghai, China; ^2^Ministry of Education-Shanghai Key Laboratory of Children’s Environmental Health, Xin Hua Hospital Affiliated to Shanghai Jiao Tong University School of Medicine, Shanghai, China; ^3^Shanghai Jiao Tong University School of Medicine, Shanghai, China; ^4^Department of Obstetrics and Gynecology, East Hospital, Tongji University School of Medicine, Shanghai, China; ^5^Obstetrics and Gynecology Hospital, Fudan University, Shanghai, China; ^6^Department of Pharmacology, Zhejiang University Medical School, Hangzhou, China

**Keywords:** fetal growth restriction, placenta, immune imbalance, fetal-reprogramming, integration analysis

## Abstract

Fetal growth restriction (FGR) is a common pregnancy complication and a risk factor for infant death. Most patients with FGR have preeclampsia, gestational diabetes mellitus, or other etiologies, making it difficult to determine the specific molecular mechanisms underlying FGR. In this study, an integrated analysis was performed using gene expression profiles obtained from Gene Expression Omnibus. Differentially expressed genes (DEGs) between healthy and FGR groups were screened and evaluated by functional enrichment and network analyses. In total, 80 common DEGs (FDR < 0.05) and 17 significant DEGs (FDR < 0.005) were screened. These genes were enriched for functions in immune system dysregulation in the placenta based on Gene Ontology and Kyoto Encyclopedia of Genes and Genomes pathway analyses. Among hub genes identified as candidates for FGR and fetal reprogramming, *LEP*, *GBP5*, *HLA–DQA1*, and *CTGF* were checked by quantitative polymerase chain reaction, immunohistochemistry, and western blot assays in placental tissues. Immune imbalance could cause hypoxia environment in placenta tissues, thus regulating the fetal-reprogramming. A significant association between *CTGF* and *HIF-1α* levels was confirmed in placenta tissues and HTR8 cells under hypoxia. Our results suggest that an immune imbalance in the placenta causes FGR without other complications. We provide the first evidence for roles of *CTGF* in FGR and show that *CTGF* may function via *HIF-1α*-related pathways. Our findings elucidate the pathogenesis of FGR and provide new therapeutic targets.

## Introduction

Fetal growth restriction (FGR) is a common pregnancy complication characterized by the failure of the fetus to reach its optimal growth potential. FGR is associated with infant morbidity and mortality (Garite et al., 2004) as well as general developmental delay in both intellectual and physical (Chen et al., 2016). Although fetuses with FGR could catch up in early life, they still have a higher risk for chronic metabolic problems as adults, including type II diabetes, insulin resistance, metabolic syndrome, and cardiovascular diseases (Gatford et al., 2010; Morrison et al., 2016; Darendeliler, 2019). A number of factors could contribute to the development of FGR, including infections, drug abuse, immune disorders, hypertension, as well as anatomical factors (Adams Waldorf and McAdams, 2013; Ankumah and Sibai, 2017; Herrera et al., 2017; Sebastiani et al., 2018). However, the exact etiology remains elusive. The roles of many transcripts and hormones in the placenta in FGR have been evaluated (Okamoto et al., 2006; Hashimoto et al., 2016; Ding and Cui, 2017). However, previous studies have included patients with multiples clinical diseases, including preeclampsia, gestational diabetes mellitus, and twin births (Deyssenroth et al., 2017; Awamleh et al., 2019; Gibbs et al., 2019; Majewska et al., 2019), and studies focusing exclusively on patients with FGR are urgently needed to provide insight into the basic pathogenesis.

The placenta plays an important role in the progression of FGR owing to its influence on fetal growth (Zhu et al., 2016). Placental dysfunction is thought to be a predominant cause of FGR, and late FGR (≥32 weeks) presents mild hypoxia with slight deficiencies in placentation (Nardozza et al., 2017). Generally, a placenta develops in a hypoxic environment associating with vascular remodeling, metabolic changes, oxidative stress, and mitochondrial dysfunction. In early pregnancy, a hypoxic environment stimulating trophoblast cells’ differentiation and vascular remodeling is essential to placentation and fetal development (Soares et al., 2017). Chronic hypoxia contributes to mitochondrial dysfunction as an underlying cause of cellular mechanisms contributing to late FGR (Song et al., 2019). What’s more, hypoxia is also a consequence of placental dysfunction, and low oxygen tension in the cord blood adversely affects fetal development (Soares et al., 2017). Hypoxia-inducible protein HIF-1α, the central factor of the cellular response to hypoxia, is closely related to the development of FGR and fetal-reprogramming (Myatt, 2006). However, the molecular pathway involving HIF-1α signal in FGR placenta metabolism is not clear, which needs to be further studied and worth exploring.

In this study, significant differentially expressed genes (DEGs) related to FGR were identified by transcriptome-level analyses of placental tissues. Publicly available message RNA (mRNA) profiles were downloaded from Gene Expression Omnibus (GEO) and divided into an FGR group and a healthy control group for the identification of DEGs. Significant DEGs obtained from multiple GEO datasets were further verified by quantitative polymerase chain reaction (qPCR), western blot, and immunohistochemistry (IHC) using placental tissues. Additionally, hypoxia directly or indirectly regulates several genes involved in cell differentiation; correlation analyses were performed for *HIF-1α* and DEGs to reveal undiscovered gene regulations for interpreting links between the hypoxic environment and fetal development.

## Materials and Methods

### Data Collection, Processing and Differentially Expressed Gene Screening

For mRNA profiles for placentas in six datasets were downloaded from the GEO repository. GSE98224, GSE75010, GSE100415, and GSE24129 were generated using the same platform, the GPL6244 [HuGene-1_0-st] Affymetrix Human Gene 1.0 ST Array [transcript (gene) version]. GSE10588 and GSE30186 were obtained using GPL2986 ABI Human Genome Survey Microarray Version 2, and GPL10558 was obtained using the Illumina HumanHT-12 V4.0 Expression BeadChip. To minimize interference from multiple conditions, mRNA profiles with descriptions including preeclampsia, large for gestational age, and preterm were excluded. Selected profiles were collected as GSM cohort and assigned to a healthy group or FGR group for subsequent analyses. The clinical characteristics of the GSM cohort are listed in the Supplementary Materials (GSM_profile_information.xls). GSM number is a data number for mRNA array data.

Background correction and quantile normalization of raw data were performed using the SVA package in *R* (version 3.6.1) (Johnson et al., 2007). The limma package in *R* was utilized to screen DEGs (Ritchie et al., 2015).^1^ Probes were annotated and an approximately normal distribution was obtained by log2 transformation. Differentially expressed genes screened with the thresholds FDR < 0.05 and | log_2_ (Fold Change)| > 0.5 were defined as common DEGs, while thresholds for significant DEGs were FDR < 0.005 and | log_2_ (Fold Change)| > 0.7. A heatmap and volcano plot were drawn using *R*.

### Bioinformatics Analysis

A Kyoto Encyclopedia of Genes and Genomes (KEGG) pathway enrichment analysis of common DEGs was performed to discover pathways that were closely associated with FGR using the ‘‘GeneAnswers’’ package.^2^ A Gene Ontology (GO) enrichment analysis was performed using the ‘‘GOstats’’ package^3^ to evaluate the functional and biological significance of DEGs. Protein--protein interaction (PPI) network information for common DEGs was obtained using the Search Tool for the Retrieval of Interacting Genes (STRING).^4^ A weighted gene co-expression network analysis (WGCNA) was performed using the WGCNA R package (Langfelder and Horvath, 2008), and networks and modules for the top 15% of genes with the largest standard deviations were obtained and visualized. Soft power thresholds (cutoff of powers = 4, scale-free *R*^2^ = 0.86) were used to generate modules. The PPI network and WGCNA results were visualized using Cytoscape (version 3.5.1), and a value of *P* < 0.05 was considered statistically significant referring to the node number.

### Study Population

Twenty-seven patients with FGR (without other pregnancy diseases) and 40 healthy pregnant women were recruited from January 2018 to December 2019 at the Obstetrics and Gynecology Hospital of Fudan University and Xin Hua Hospital Affiliated to Shanghai Jiao Tong University School of Medicine, Shanghai, China. The study was approved by the Ethics Committee of the hospital (approval number: XHEC-D-2020-116). All recruited patients provided signed, informed consent prior to the collection of placental tissues. Initial recruitment for participates was performed on pregnant women at gestational age about 28 weeks, and cases of FGR were primarily screened using fetoplacental Doppler. With the progress of the pregnancy, some FGR cases were relieved. Finally, only the newborn has FGR at delivery donated their placental tissues. Thus placenta donors recruited in our study also belong to SGA. Donors met the following criteria: (1) 20–35 years of age and a gestational age of not less than 37 weeks; (2) singleton pregnancy; (3) non-smoking and non-drinking; and (4) no other pregnancy complications or genetic deficiency now or previously.

### Sample Processing

The maternal decidua and fetal chorion on the surface of the placenta were removed and discarded before sampling. Due to the complex and heterogeneous characteristics of the placenta, small pieces of tissues were cut from six different regions of the placenta to avoid the impact of the spatial discrepancy, and each region was 4–6 cm away from the terminal of the umbilical cord. Tissue samples were taken from placentas within 30 min after delivery, fixed in formalin for hematoxylin–eosin (HE) staining and IHC, or frozen immediately in liquid nitrogen and stored at –80°C for RNA extraction. Total RNAs were isolated from placental tissues using an RNeasy Plus Universal Kit (Cat. No. 74134; Qiagen, Hilden, Germany) according to the manufacturer’s instructions. RNA quantity and quality were assessed using an ultraviolet spectrophotometer (ND-1000; NanoDrop Technologies, Wilmington, DE, United States) and an electrophoresis device (Tanon 3500; Tanon, Shanghai, China), respectively.

### Quantitative Polymerase Chain Reaction

Total RNA was reverse transcribed using a PrimeScript^TM^ RT Reagent Kit containing a gDNA eraser (RR047A; Takara, Kusatsu, Japan) according to the manufacturer’s instructions. Briefly, 1 μg total RNA of each sample was reverse transcribed, and cDNA samples were checked about concentrations and pre-diluted to a concentration of about 100 ng/μl for further qPCR assay. The qPCR analysis was performed by following the MIQE guidelines (Bustin et al., 2009). Gene expression levels were detected by qPCR using the SYBR-Green PCR Kit (FP205; Tiangen, Beijing, China) and an Applied Biosystems Q7 Real-time PCR instrument (Applied Biosystems, Carlsbad, CA, United States). PCR was conducted under the following cycling conditions: 95°C for 15 min for activation, followed by 40 cycles of 95°C for 10 s, 58°C for 30 s, and 72°C for 30 s. The relative gene expression levels of candidate genes were normalized to the level of *GAPDH* and calculated by the comparative CT (2^–ΔΔ^*^*Ct*^*) method. The sequences of primers are presented in Supplementary Table 1. The amplification efficiencies of the genes ranged from 90 to 115%. The melting curves of tested genes indicated that the amplification was specific (Supplementary Figure 1). Also, genes showed a consistent expression level using the reference gene of either GAPDH or Beta-action (Figure 4C and Supplementary Figure 2).

**FIGURE 1:**
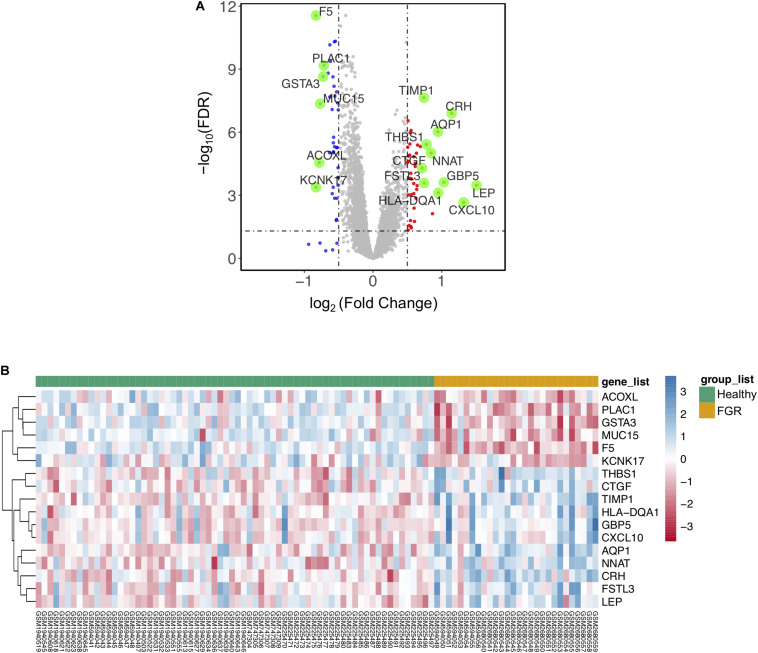
Significant differentially expressed genes (DEGs) based on an integrated analysis. **(A)** Volcano map for DEGs; red dots and blue dots indicate genes that were up-regulated and down-regulated (FDR < 0.05) in the fetal growth restriction (FGR) group, respectively. The green dots indicate significant DEGs (FDR < 0.005). **(B)** Two-way hierarchical clustering map of significant DEGs for FGR; upper bar in green indicates the healthy group and yellow indicates the FGR group.

**FIGURE 2:**
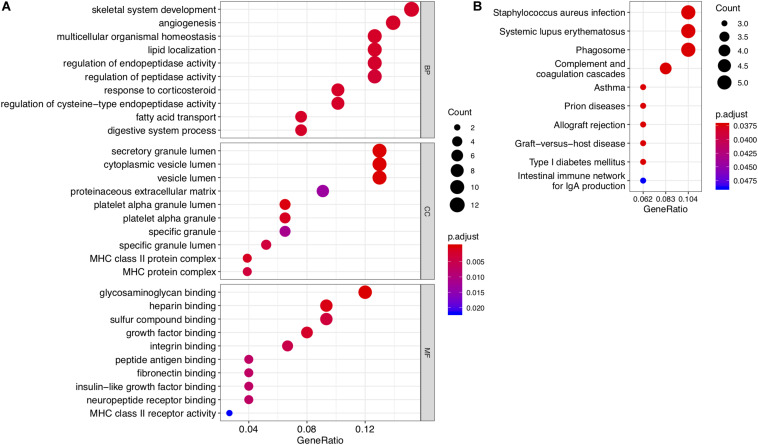
Results of GO enrichment and KEGG pathway analyses of DEGs. **(A)** Top 10 enriched GO terms for common DEGs with the smallest *P-*values in the three broad categories: biological process, molecular function, and cellular component. **(B)** Top 10 KEGG pathways for common DEGs.

**FIGURE 3:**
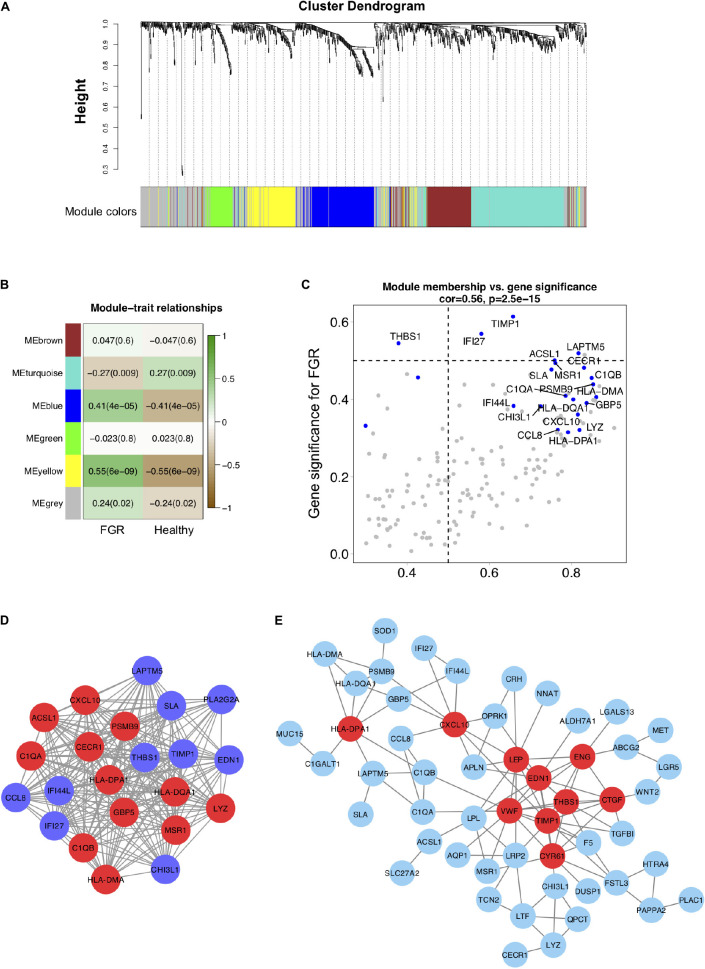
Co-expression modules generated by weighted gene co-expression network analysis and Protein–protein interaction network analyses. **(A)** Cluster dendrogram of 96 samples with eligible data. Five modules (turquoise, grey, blue, brown, green, and yellow) were extracted. The grey color represents genes that cannot be classified into any module. **(B)** Heatmap of the correlation between DEGs in co-expression modules. **(C)** Scatter plot of DEGs in the blue module. **(D)** The blue co-expression module generated by WGCNA contained the most significant DEGs. **(E)** DEGs were used to generate a PPI network using the STRING online database; hub genes are indicated in red.

**FIGURE 4:**
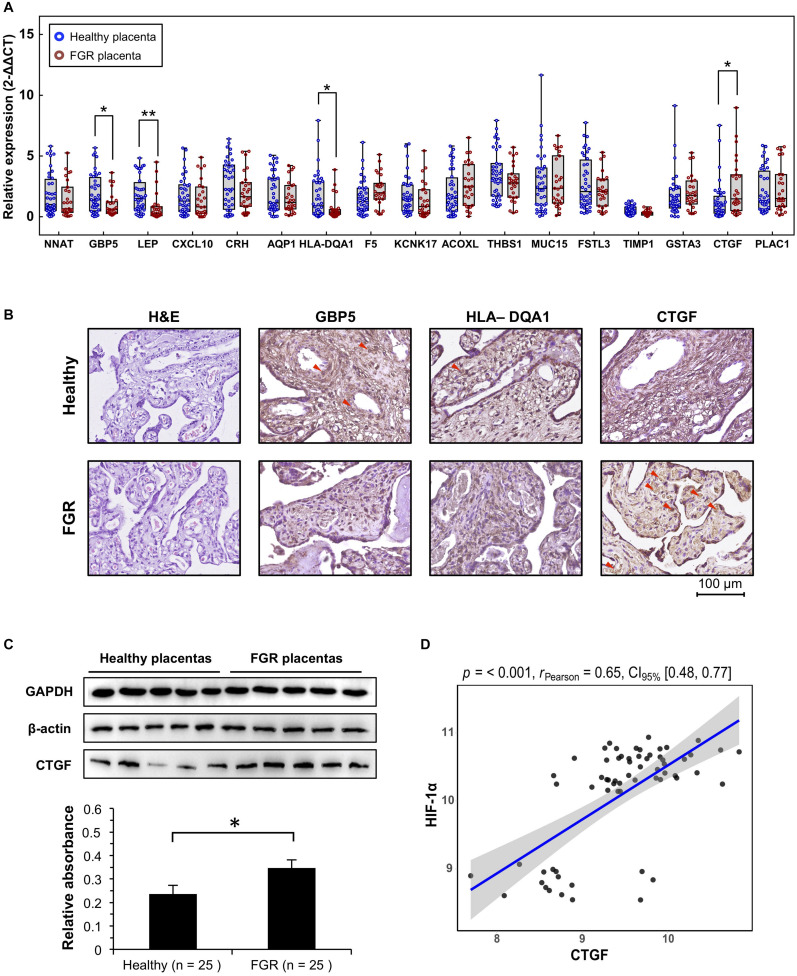
Verification of significant DEGs at the transcript and protein levels. **(A)** Quantitative polymerase chain reaction (qPCR) analysis of placental tissues to verify the transcript levels of significant DEGs. **(B)** Representative immunohistochemistry results for placenta tissues to confirm candidate DEGs. **(C)** CTGF protein, quantified using western blotting. Representative five blots are shown. **(D)** The expression level of *CTFG* in GEO microarray data was significantly associated with that of *HIF-1α.* ^∗^*P* < 0.05, ^∗∗^*P* < 0.005.

### Hematoxylin–Eosin Staining and Immunohistochemistry of Placental Tissues

Placentas maintained in formalin were paraffin-embedded and sliced, followed by histological grading by HE staining. IHC was also performed on sections to verify the results for genes at the protein level, as described by Magaki et al. (2019), with a few modifications. Briefly, After blocking with 10% non-immune serum, the sections were incubated with antibody to GBP5 (13220-1-AP; Proteintech, Chicago, IL, United States; 1:150 dilution), HLA–DQA1 (16918-1-AP; Proteintech; 1:100 dilution), and CTGF (23936-1-AP; Proteintech; 1:200 dilution) at room temperature for 2 h, followed by incubation with the secondary antibody (Santa Cruz Biotechnology, United States) for 1 h. After incubation in a streptavidin–peroxidase conjugate, the antibody complexes were visualized with diaminobenzidine tetrahydrochloride chromogen for 10 s. Stained tissue sections were observed using an optical microscope (Olympus^TM^ BX50, Tokyo, Japan).

### Cell Culture and Hypoxia Treatment

The trophoblast cell line HTR8/SVneo (HTR8) was provided as a gift by the International Peace Maternity & Child Health Hospital of China Welfare Institute (IPMCH). Cells were verified by STR profiling and cultured in modified RPMI1640 medium (without calcium nitrate, with L-glutamine) (Hyclone, Logan, UT, United States) supplemented with 10% fetal bovine serum (Biowest, Nuaillé, France) and 1% penicillin–streptomycin (Gibco, Carlsbad, CA, United States). Cells were cultured on tissue culture dishes (Thermo Fisher Scientific, Waltham, MA, United States) at 37°C in a 1% O_2_ incubator to simulate hypoxia, while a condition of 10% O_2_ has been applied for the routine culture. The HTR8 cell line was used as a model for observing the gene functions of placental extravillous trophoblast in the current study.

### Western Blot Analysis

Cells cultured in a hypoxic environment were collected at 0 and 48 h. Then, western blotting was performed to determine the expression level of *CTGF*. Briefly, cells were washed with cold PBS and lysed in RIPA buffer (Beyotime, Shanghai, China) on ice. Tissues were crumbled before lysing. The supernatant of each sample was collected and quantified by a commercial Bicinchoninic Acid Kit (P0012S; Beyotime Biotechnology, China) for protein determination. Then 20 μg of protein for each sample was separated by 10% SDS/PAGE and transferred to PVDF membranes (Roche, Basel, Switzerland). After blocking in 5% skim milk/TBS–Tween-20 (1%) and incubating with primary antibodies against CTGF (23936-1-AP; Proteintech; 1:1,000 dilution), HIF-1α (20960-1-AP; Proteintech; 1:1,000 dilution), GAPDH (10494-1-AP; Proteintech; 1:20,000 dilution) or β-actin (20536-1-AP; Proteintech; 1:2,000 dilution), membranes were incubated with secondary antibodies and visualized using a chemiluminescence-based detection system (Pierce Biotechnology, Rockford, IL, United States). The intensity of the protein spot was analyzed using densitometry normalized to GAPDH expression.

### Statistical Analyses

Data are expressed as mean values and standard deviations. The Student’s *t*-test was applied to normally distributed data for comparisons between groups, and the Chi-square test was used to evaluate the sex ratio of newborns. A Pearson correlation assay was applied to the microarray data and qPCR to obtain the correlation coefficients for the relationships between the expression level of *HIF-1α* and those of significant DEGs. A value of *P* < 0.05 was considered statistically significant.

## Results

### Identification of DEGs in FGR-Related GSM Profiles

To identify genes associated specifically with FGR without other pregnancy complications, 96 GSM profiles from six GEO datasets were downloaded, screened, and divided into a healthy group (68 healthy subjects) and an FGR group (28 FGR subjects). GSM profiles were successfully normalized (Supplementary Figure 3). A total of 80 common DEGs (FDR < 0.05) were screened and used to generate a heatmap (Supplementary Figure 4), and 17 genes with significant differences (FDR < 0.005) were considered significant DEGs (Table 1). A volcano plot and heat map of significant DEGs are shown in Figures 1A,B, respectively. A list of common DEGs is provided in the Supplementary Materials (DEGs.xls). Clinic characteristics were briefly summarized in Supplementary Table 2.

**TABLE 1:** Significant differentially expressed genes (DEGs) in placental tissues of patients with FGR.

Gene name	FDR	logFC	Description
*F5*	2.80E−12	–0.830	Coagulation Factor V
*PLAC1*	6.73E−10	–0.715	Placenta Enriched 1
*GSTA3*	2.28E−09	–0.726	Glutathione *S*-Transferase Alpha 3
*TIMP1*	2.28E−08	0.740	TIMP Metallopeptidase Inhibitor 1
*MUC15*	4.45E−08	–0.770	Mucin 15, Cell Surface Associated
*CRH*	1.27E−07	1.143	Corticotropin Releasing Hormone
*AQP1*	9.50E−07	0.944	Aquaporin 1 (Colton Blood Group)
*THBS1*	3.76E−06	0.777	Thrombospondin 1
*NNAT*	9.86E−06	0.844	Neuronatin
*ACOXL*	2.81E−05	–0.782	Acyl-CoA Oxidase Like
*CTGF*	5.08E−05	0.712	Cellular Communication Network Factor 2
*GBP5*	0.000242	1.030	Guanylate Binding Protein 5
*FSTL3*	0.000261	0.743	Follistatin Like 3
*LEP*	0.000338	1.506	Leptin
*KCNK17*	0.000415	–0.830	Potassium Two Pore Domain Channel Subfamily K Member 17
*HLA-DQA1*	0.000759	0.950	Major Histocompatibility Complex, Class II, DQ Alpha 1
*CXCL10*	0.00217	1.319	C–X–C Motif Chemokine Ligand 10

### Functional Enrichment Analysis of Common DEGs

To investigate the functions and biological pathways involving common DEGs, GO enrichment, and KEGG analyses were performed. The 10 most highly significant GO terms, including biological processes, molecular functions, and cellular components, are summarized in Supplementary Table 3. As shown in Figure 2A, the common DEGs were enriched for various molecular functions (e.g., glycosaminoglycan binding), cellular components (e.g., secretory granule lumen), and biological processes (e.g., skeletal system development). The top 10 enriched KEGG pathways are summarized in Figure 2B and Supplementary Table 4. The common DEGs were mainly involved in immune and inflammatory pathways, such as *Staphylococcus aureus* infection, asthma, and systemic lupus erythematosus.

### Interactome Networks of Genes Generated by WGCNA and PPI

To evaluate interactions among genes related to FGR, a gene co-expression network analysis using WGCNA was performed with the top 15% of genes (1,083) with the largest standard deviations (Figure 3A). Five modules were extracted (shown in different colors in Figure 3). According to an analysis of module–trait relationships (Figure 3B), three modules (shown in turquoise, blue, and yellow) with *P-*values of <0.05 were identified as significant. Significant DEGs acting as hub genes in significant modules are key candidates for FGR and are listed in Table 3. The blue module shown in Figures 3C,D contained the most hub genes classified as significant DEGs. We defined the term “specific DEGs” for the significant DEGs acting as hubs in modules and networks, indicating their important roles for the pathology of FGR placentas. The module components and gene co-expression networks for the other two significantly modules (i.e., yellow and turquoise) are presented in Supplementary Figure 5.

**TABLE 2:** Clinical characteristics of healthy individuals and patients with FGR.

Variables	Healthy	FGR
Age (years)	29.13 ± 3.39	29.18 ± 3.82
Gestational age (weeks)	39.10 ± 2.63	38.04 ± 2.11
Maternal ethnicity	Chinese	Chinese
Tissue type	Placenta	Placenta
Previous IUGR	No	No
Previous miscarriage	No	No
Previous hypertensive pregnancy	No	No
Mode proteinuria	No	No
HELLP diagnosis	No	No
IUGR diagnosis	No	Yes
Chorioamnionitis diagnosis	No	No
Infant gender (Male/Female)	22/18	11/16
Infant weight (g)	3,340.43 ± 330.28	2,260.00^∗^ ± 332.56
Aparg-score 1 min	9.55 ± 0.50	9.48 ± 0.50
Aparg-score 5 min	9.88 ± 0.33	9.48 ± 0.50
Mode of delivery (Cesarean section/Eutocia)	19/21	11/20

*^∗^*P* < 0.01.*

**TABLE 3:** Eight specific DEGs identified as hub genes in the WGCNA or PPI analysis were determined to be key genes for the pathogenesis of FGR.

Gene name	qPCR-verified	WGCNA modules	PPI network
*TIMP1*	×	×	○
*MUC15*	×	“turquoise”	×
*THBS1*	×	×	○
*CTGF*	○	×	○
*GBP5*	○	“blue”	×
*LEP*	○	×	○
*HLA-DQA1*	○	“blue”	×
*CXCL10*	×	“blue”	○

Protein–protein interaction networks for 80 common DEGs were generated using the STRING database. *TIMP1*, *VWF*, *EDN1*, *LEP*, *THBS1*, *CXCL10*, *CYR61*, *CTGF*, *ENG*, and *HLA–DPA1* were significant (*P* < 0.05) in the PPI networks (Figure 3E). *TIMP1*, *LEP*, *THBS1*, *CXCL10*, and *CTGF* identified as specific DEGs are listed in Table 3 and are key genes associated with FGR.

### Verification of Significant DEGs in Placental Tissues

Excluding the weight of the fetus, there were no significant differences in age, fetal gender, pregnancy age, and other characteristics between the two groups (Table 2). Quantitative PCR results suggested that *GBP5* (*P* = 0.009, Fold Change = 1.979), *LEP* (*P* = 0.002, Fold Change = 2.359), *HLA–DQA1* (*P* = 0.015, Fold Change = 2.404), and *CTGF* (*P* = 0.018, Fold Change = 0.529) were significantly related to FGR (Figure 4A). LEP is widely known about it relationship with FGR pathogenesis. *GBP5* and *HLA–DQA1* are newly defined genes associated with FGR. A previous study has reported an association between *CTGF* and FGR in serum and placenta (Oh et al., 2009); however, they used a very few idiopathic FGR placenta samples (*n* < 10). These results were confirmed by IHC analyses of *CTGF*, *GBP5*, and *HLA–DQA1* in placental tissues (Figure 4B). Five samples in each group were used for validation. The *CTGF* presented a significant difference (three strong and two moderate in the FGR group, two moderate and three weak in the healthy group). The *GBP* presented a significant difference (two weak and three moderate in the FGR group, one moderate and four strong in the healthy group). The *HLA–DQA1* presented differences but not significant (one weak, three moderate and one strong in the FGR group, three moderate and two strong in the healthy group). The western bot assay further confirmed the up-regulation of *CTGF* in FGR placental tissues (Figure 4C), which was consistent with the qPCR assay. Pearson correlation analysis on GEO microarray data showed a significant correlation of *HIF-1α* and *CTGF* (*R* = 0.65, *P* < 0.001), suggesting their cooperation in the pathology of placenta tissues (Figure 4D).

### DEGs Associated With the Hypoxic Microenvironment

**FIGURE 5:**
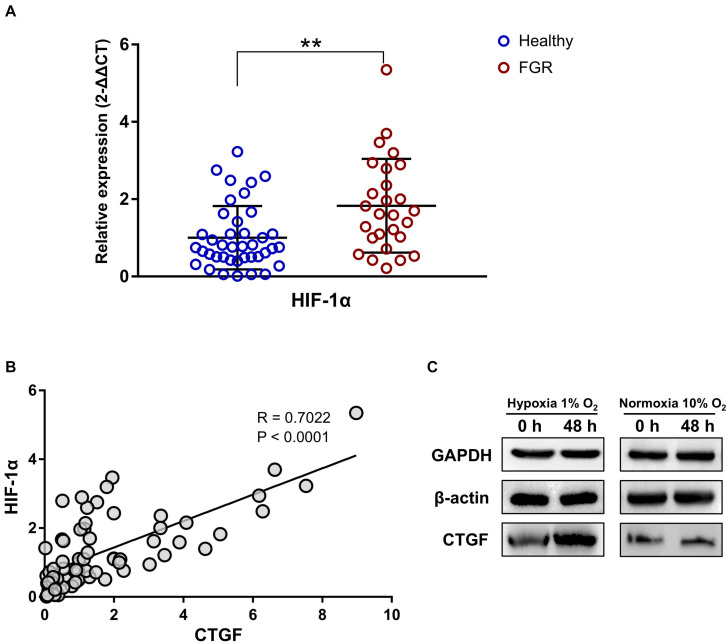
The expression level of *CTFG* was related to the “hypoxic” environment and was associated with the level of *HIF-1α*. **(A)** The significant up-regulation of *HIF-1α* was revealed by a qPCR assay of placental tissues. **(B)** The expression level of *CTFG* was significantly associated with that of *HIF-1α.* **(C)** Compared to the normoxic control, *CTFG* levels were higher in the “hypoxic” environment. ^∗∗^*P* < 0.005.

As determined by qPCR, *HIF-1α* was significantly up-regulated in FGR placentas (*P* = 0.001, Fold Change = 0.549, Figure 5A). This result may reflect the more serious hypoxic environment for FGR placentas than for normal placentas. Pearson correlation coefficients were obtained for the relationships between mRNA levels of *HIF-1α* and significant DEGs. *CTGF* was significantly associated was *HIF-1α* (*R* = 0.702, *P* < 0.0001, Figure 5B). A western blot assay was conducted using the HTR8 placental trophoblast cell line to confirm the association between *CTGF* expression and a hypoxic environment. In HTR8 cells cultured in a hypoxic environment, *CTGF* expression levels were up-regulated (Figure 5C), indicating that it may be involved in *HIF-1α*-related regulatory pathways under hypoxia.

## Discussion

The placenta, as a source of peptides and steroid hormones, directly supports fetal development. More importantly, it provides an immune interface between the mother and the fetal allograft (Myatt, 2006). In this study, we identified 17 significant DEGs (Table 1) and 8 specific hub genes (Table 3) associated with FGR based on an integrated analysis of a GSM cohort. According to GO and KEGG pathway enrichment analyses (Supplementary Tables 3 and 4), the DEGs in FGR placentas were mainly enriched for functions and pathways related to an immunologic imbalance. In addition, the results for newly identified *GBP5*, *HLA–DQA1*, and *CTGF* were verified at the protein (IHC and western blot) and transcript (qPCR) levels, and CTGF was found as a key gene associated with idiopathic FGR. In addition, the expression of *CTGF* is closely associated with *HIF-1α*. These results improve our understanding of the pathogenesis of FGR without other pre-gestational diseases and offer potential therapeutic targets for incurable cases. For example, therapies targeting related pathways might relieve the disorder of intracellular metabolism brought by the hypoxia environment and the immunologic imbalance, which were the two main phenotypes of the case of unexplained FGR.

Epidemiological evidence in FGR pregnancies has linked a low birth weight to fetal programming. The differential expression levels of *LEP, GBP5, HLA–DQA1*, and *CTGF* were confirmed by qPCR (Figure 4A) and these loci were identified as hub genes in networks and thereby likely play key roles in FGR (Figure 3D,E and Table 3). *LEP*, the most highly significantly down-regulated gene in FGR placentas, encodes leptin, found in the circulation. Leptin plays a major role in the regulation of energy homeostasis and obesity and is involved in endocrine functions, including the regulation of immune and inflammatory responses, angiogenesis, reproduction, and bone formation (Bodary et al., 2007; Steinbrekera and Roghair, 2016). Maternal and fetal leptin levels are correlated with FGR originating from damaged placental function (Zareaan et al., 2017). *CTGF*, the most significant up-regulated gene, encodes connective tissue growth factor. This factor is related to platelet-derived growth factor and plays roles in chondrocyte proliferation and differentiation as well as cell adhesion (Fujisawa et al., 2008; Kiwanuka et al., 2011). Additionally, as shown in Supplementary Table 3, *CTGF* contributed to the top 5 GO terms in the molecular function category (i.e., glycosaminoglycan binding, heparin binding, growth factor binding, sulfur compound binding, and integrin binding) and the top 2 GO terms in the biological process category (i.e., response to corticosteroid and skeletal system development), suggesting that it influences the metabolism and immune function in fetal development. Significantly down-regulated *GBP5* encodes guanylate binding protein 5, which is related to the innate immune system and the NF-κB pathway. Previous studies have suggested that *GBP5* is involved in the mechanism underlying FGR caused by post-natal innate immune alterations. An altered innate immune system is associated with FGR based on the increased susceptibility to infections in the postnatal period (Amdi et al., 2020). Additionally, the NF-κB pathway contributes to vascular growth in the developing fetal lung, and the maintenance of endothelial NF-κB activation may be useful in FGR marked by disrupted angiogenesis (Dodson et al., 2018). The significantly down-regulated gene *HLA–DQA1* encodes a membrane-anchored protein for presenting peptides derived from extracellular proteins. It is mainly expressed in lymphocytes, dendritic cells, and macrophages; these antigen-presenting cells show altered plasticity in FGR placentas (Freitag et al., 2014; Liu et al., 2015; Chu et al., 2019). In addition, *HLA–DQA1* was involved in 8 of the top 10 KEGG pathways (see Supplementary Table 3), indicating that it is closely associated with FGR and fetal programming via immunologic processes.

*HIF-1α* activates gene transcription in response to changes in oxygen concentrations and accumulates in FGR placentas (Tal et al., 2010). *HIF-1α* transcription levels were significantly up-regulated in this study. A previous study has demonstrated that maternal inflammation-induced FGR is associated with increased placental *HIF-1α* (Robb et al., 2017); thus, we hypothesize that the hypoxic microenvironment presenting in most FGR placentas (Figure 5A) could be attributed to immune dysfunction. This inference is supported by the results of our bioinformatics analyses. The hypoxic environment induced oxidative DNA damage in the placenta in FGR (Kimura et al., 2013), which may explain the link between the epidemiological characteristics of FGR and fetal reprogramming. In addition, we obtained the first evidence for a correlation between the expression levels of *CTGF* and *HIF-1α*. Previous reports have demonstrated that *CTGF* could be affected by a hypoxic environment (Rimon et al., 2008). Our western blot assay using HTR8 cells confirmed the accumulation of *CTGF* under hypoxia, further suggesting CTGF contributes to hypoxia-related pathways. This finding reveals a common mechanism linking the hypoxic environment to fetal programming as well as the pathogenesis of FGR.

Unlike gestational diabetes mellitus- and preeclampsia-induced FGR, which are mainly explained by metabolic syndrome or hypertensive disorder (Ornoy, 2011; Gibbs et al., 2019), our results suggested that the main cause of FGR without other complications is probably abnormal immune functions in the placenta. The novel key genes (*GBP5, HLA–DQA1*, and *CTGF*) provide insight into the mechanism underlying FGR development and fetal programming and provide therapeutic targets for improving prognosis. Despite the important discoveries, our study had some limitations. Research revealing biomarkers depends on population-based analyses; however, FGR placentas without other diseases are not easy to collect and thus a larger sample size could improve the discovery of specific FGR-related genes. Furthermore, experiments using cell and animal models are necessary to confirm the newly identified genes and clarify their precise roles.

## Data Availability Statement

Publicly available datasets were analyzed in this study. This data can be found here: Six datasets were downloaded from the GEO repository: GSE98224, GSE75010, GSE100415, GSE24129, GSE10588, and GSE30186.

## Ethics Statement

The studies involving human participants were reviewed and approved by The study was approved by the Ethics Committee of the hospital (approval number: XHEC-D-2020-116). The patients/participants provided their written informed consent to participate in this study.

## Author Contributions

XW and X-rX: conceptualization, funding acquisition, and review and editing. HZ and YZ: data curation and analysis. HZ and J-rZ: investigation. J-xW: resources. XW: data visualization and writing draft. J-rZ and X-rX: supervision. CT: validation. All authors contributed to the article and approved the submitted version.

## Conflict of Interest

The authors declare that the research was conducted in the absence of any commercial or financial relationships that could be construed as a potential conflict of interest.

## Footnotes

1 https://bioconductor.org/packages/release/bioc/html/limma.html

2 http://www.bioconductor.org/packages/release/bioc/html/GeneAnswers.html

3 http://www.bioconductor.org/packages/release/bioc/html/GOstats.html

4 http://www.string-db.org/
