# Supplementary material for: Integrated Analysis of Key Genes and Pathways Involved in Fetal Growth Restriction and Their Associations With the Dysregulation of the Maternal Immune System
Source: Front Genet. 2021 Jan 8;11:581789. doi: 10.3389/fgene.2020.581789 (PMC7873903; doi:10.3389/fgene.2020.581789)
Supplement: Supplementary file 1 [file Data_Sheet_1.zip › SupplementalData/SupplementalTables_revised.docx]

Table S1 Designed primers for detecting gene expression levels

| Gene name | Product size (bp) |  | Sequences of Primers |
| --- | --- | --- | --- |
| *LEP* | 199 | **F:** | 5' - CATCAAGACAATTGTCACCAGG - 3' |
|  |  | **R:** | 5' - GTCGTTGGATATTTGGATCACG - 3' |
| *CXCL10* | 161 | **F:** | 5' - CTCTCTCTAGAACTGTACGCTG - 3' |
|  |  | **R:** | 5' - ATTCAGACATCTCTTCTCACCC - 3' |
| *CRH* | 145 | **F:** | 5' - GCATGGGAGAGGAGTACTTC - 3' |
|  |  | **R:** | 5' - AACACGCGGAAAAAGTTGG - 3' |
| *GBP5* | 117 | **F:** | 5' - CCCTAAGGCCAAAGCAAG - 3' |
|  |  | **R:** | 5' - GGTGAGCAGGTAAGTCAAAGATAA - 3' |
| *HLA-DQA1* | 114 | **F:** | 5' - GAGGTGAAGACATTGTGGCT - 3' |
|  |  | **R:** | 5' - CGTAGAACTGCTCATCTCCAT - 3' |
| *AQP1* | 100 | **F:** | 5' - GGACCAAGATTTACCAATTCAC - 3' |
|  |  | **R:** | 5' - CACCTCCCATCATGCTCTG - 3' |
| *NNAT* | 91 | **F:** | 5' - CGGCTGGTACATCTTCCGC - 3' |
|  |  | **R:** | 5' - TGTCCCTGGAGGATTTCGAAA - 3' |
| *F5* | 105 | **F:** | 5' - AAGCCCTTGAGCATCCATC - 3' |
|  |  | **R:** | 5' - CACAGCGTCGTCCATCTTCT - 3' |
| *KCNK17* | 185 | **F:** | 5' - ATCTCATGCAGCAGGGAGTAAA - 3' |
|  |  | **R:** | 5' - GAAGTAGAAGCCCTCTGTGTAG - 3' |
| *ACOXL* | 81 | **F:** | 5' - GATGGAAAATCGAATCTCAGGC - 3' |
|  |  | **R:** | 5' - TGAAGCATAACAACATCGTCAC - 3' |
| *THBS1* | 182 | **F:** | 5' - TTTGACATCTTTGAACTCACCG - 3' |
|  |  | **R:** | 5' - AGAAGGAGGAAACCCTTTTCTG - 3' |
| *MUC15* | 161 | **F:** | 5' - AGTTCTGCGATTAGACAATG - 3' |
|  |  | **R:** | 5' - ATACAGAAGTACGAAGTGGAG - 3' |
| *FSTL3* | 153 | **F:** | 5' - CAACAACAACGTCACCTACATC - 3' |
|  |  | **R:** | 5' - ACGAAGTTCTCTTCCTCTTCTG - 3' |
| *TIMP1* | 119 | **F:** | 5' - CATCACTACCTGCAGTTTTGTG - 3' |
|  |  | **R:** | 5' - TGGATAAACAGGGAAACACTGT - 3' |
| *GSTA3* | 114 | **F:** | 5' - TGCCTTCGAAAAAGTGTTACAG - 3' |
|  |  | **R:** | 5' - GAGTCAAGCTCTTCCACATAGT - 3' |
| *PLAC1* | 133 | **F:** | 5' - CTTTCATGAACTACACTTGGGC - 3' |
|  |  | **R:** | 5' - GCTGTAGATAACCATGTCCTGA - 3' |
| *CTGF* | 196 | **F:** | 5' - ATTCTGTGGAGTATGTACCGAC - 3' |
|  |  | **R:** | 5' - GTCTCCGTACATCTTCCTGTAG - 3' |
| *GAPDH* | 112 | **F:** | 5' - GACTCATGACCACGTCCATGC - 3' |
|  |  | **R:** | 5' - AGAGGCAGGGATGATGTTCTG - 3' |

Table S2 Clinical characteristics of healthy individuals and patients with FGR in GSM profiles downloaded from GEO database.

| **Variables** | **Healthy (Count)** | **FGR (Count)** |
| --- | --- | --- |
| Age (years) | 31.91 ± 4.84 (34) | 33 ± 4.03 (20) |
| Maternal bmi | 24.67 ± 5.29 (28) | 24.24 ± 6.13 (16) |
| Maximum systolic bp | 124.71 ± 14.04 (28) | 129.63 ± 12.01 (20) |
| Maximum diastolic bp | 79.11 ± 7.84 (28) | 80.15 ± 10.64 (20) |
| Gestational age (weeks) | 38.01 ± 1.46 (28) | 37.14 ± 1.34 (20) |
| Previous IUGR | No | No |
| Other pregnancy complications | No | No |
| Infant gender (Male/Female) | 16/12 (28) | 8/12 (20) |
| Mode of delivery (Caesarean section/Eutocia) | 19/9 (28) | 8/12 (20) |

Information for some cases are missing and not offered in source data, number in brackets means the number of patients counted.

Table S3 Top 10 enriched GO terms for DEGs from the aspect of biological process, cellular component, and molecular function.

| Ontology | GO ID | Description | *P* value | Gene_symbol |
| --- | --- | --- | --- | --- |
| Biological Process | 0031960 | response to corticosteroid | 1.71E-03 | AGL/CRH/AQP1/DUSP1/ENG/CTGF/EDN1/CLDN1 |
|  | 0001501 | skeletal system development | 1.71E-03 | FBN2/TIMP1/TGFBI/CYR61/ENG/CTGF/EDN1/NPR3/LEP/PAPPA2/CHI3L1/LTF |
|  | 0001525 | angiogenesis | 1.71E-03 | C1GALT1/TGFBI/AQP1/THBS1/CYR61/ENG/CTGF/EDN1/LEP/CHI3L1/CXCL10 |
|  | 0048871 | multicellular organismal homeostasis | 1.71E-03 | APLN/SOD1/AQP1/CTGF/MET/CLDN1/OPRK1/LYZ/LTF/SERPINA3 |
|  | 0015908 | fatty acid transport | 1.71E-03 | THBS1/SLC27A2/ACSL1/EDN1/LEP/PLA2G2A |
|  | 0022600 | digestive system process | 1.71E-03 | CRH/AQP1/NPR3/LEP/OPRK1/SERPINA3 |
|  | 0010876 | lipid localization | 1.72E-03 | CRH/THBS1/MSR1/SLC27A2/ABCG2/ACSL1/EDN1/LEP/PLA2G2A/LPL |
|  | 2000116 | regulation of cysteine-type endopeptidase activity | 1.74E-03 | AQP1/IFI27/THBS1/CYR61/DHCR24/CTGF/PSMB9/LTF |
|  | 0052548 | regulation of endopeptidase activity | 2.13E-03 | TIMP1/AQP1/IFI27/THBS1/CYR61/DHCR24/CTGF/PSMB9/LTF/SERPINA3 |
|  | 0052547 | regulation of peptidase activity | 2.79E-03 | TIMP1/AQP1/IFI27/THBS1/CYR61/DHCR24/CTGF/PSMB9/LTF/SERPINA3 |
| Cellular Component | 0034774 | secretory granule lumen | 9.29E-05 | F5/AGL/TIMP1/VWF/THBS1/CHI3L1/QPCT/LYZ/LTF/SERPINA3 |
|  | 0060205 | cytoplasmic vesicle lumen | 9.29E-05 | F5/AGL/TIMP1/VWF/THBS1/CHI3L1/QPCT/LYZ/LTF/SERPINA3 |
|  | 0031983 | vesicle lumen | 9.29E-05 | F5/AGL/TIMP1/VWF/THBS1/CHI3L1/QPCT/LYZ/LTF/SERPINA3 |
|  | 0031093 | platelet alpha granule lumen | 4.22E-04 | F5/TIMP1/VWF/THBS1/SERPINA3 |
|  | 0042613 | MHC class II protein complex | 1.25E-03 | HLA-DQA1/HLA-DMA/HLA-DPA1 |
|  | 0031091 | platelet alpha granule | 1.25E-03 | F5/TIMP1/VWF/THBS1/SERPINA3 |
|  | 0035580 | specific granule lumen | 3.51E-03 | CHI3L1/QPCT/LYZ/LTF |
|  | 0042611 | MHC protein complex | 3.51E-03 | HLA-DQA1/HLA-DMA/HLA-DPA1 |
|  | 0042581 | specific granule | 1.17E-02 | SLC27A2/CHI3L1/QPCT/LYZ/LTF |
|  | 0005578 | proteinaceous extracellular matrix | 1.34E-02 | FBN2/TIMP1/TGFBI/VWF/CTGF/WNT2/CHI3L1 |
| Molecular Function | 0005539 | glycosaminoglycan binding | 6.51E-05 | EXTL2/THBS1/CYR61/ENG/CTGF/CXCL10/CCL8/LPL/LTF |
|  | 0008201 | heparin binding | 6.38E-04 | THBS1/CYR61/CTGF/CXCL10/CCL8/LPL/LTF |
|  | 0019838 | growth factor binding | 1.91E-03 | THBS1/CYR61/DUSP1/ENG/CTGF/HTRA4 |
|  | 1901681 | sulfur compound binding | 3.60E-03 | THBS1/CYR61/CTGF/CXCL10/CCL8/LPL/LTF |
|  | 0005178 | integrin binding | 4.90E-03 | TGFBI/VWF/THBS1/CYR61/CTGF |
|  | 0042605 | peptide antigen binding | 7.43E-03 | DHCR24/HLA-DQA1/HLA-DPA1 |
|  | 0001968 | fibronectin binding | 7.43E-03 | THBS1/CTGF/FSTL3 |
|  | 0005520 | insulin-like growth factor binding | 7.43E-03 | CYR61/CTGF/HTRA4 |
|  | 0071855 | neuropeptide receptor binding | 7.43E-03 | APLN/CRH/EDN1 |
|  | 0032395 | MHC class II receptor activity | 2.18E-02 | HLA-DQA1/HLA-DPA1 |

Table S4 Top 10 KEGG pathways for DEGs

| ID | Description | pvalue | Gene_symbol |
| --- | --- | --- | --- |
| hsa05150 | *Staphylococcus aureus* infection | 3.73E-02 | C1QB/HLA-DQA1/HLA-DMA/C1QA/HLA-DPA1 |
| hsa05310 | Asthma | 3.73E-02 | HLA-DQA1/HLA-DMA/HLA-DPA1 |
| hsa05020 | Prion diseases | 3.73E-02 | SOD1/C1QB/C1QA |
| hsa05322 | Systemic lupus erythematosus | 3.73E-02 | C1QB/HLA-DQA1/HLA-DMA/C1QA/HLA-DPA1 |
| hsa04610 | Complement and coagulation cascades | 3.73E-02 | F5/VWF/C1QB/C1QA |
| hsa05330 | Allograft rejection | 3.73E-02 | HLA-DQA1/HLA-DMA/HLA-DPA1 |
| hsa05332 | Graft-versus-host disease | 3.73E-02 | HLA-DQA1/HLA-DMA/HLA-DPA1 |
| hsa04145 | Phagosome | 3.73E-02 | THBS1/MSR1/HLA-DQA1/HLA-DMA/HLA-DPA1 |
| hsa04940 | Type I diabetes mellitus | 3.73E-02 | HLA-DQA1/HLA-DMA/HLA-DPA1 |
| hsa04672 | Intestinal immune network for IgA production | 4.88E-02 | HLA-DQA1/HLA-DMA/HLA-DPA1 |
